# Maternal total cell-free DNA in preeclampsia with and without intrauterine growth restriction

**DOI:** 10.1038/s41598-020-68842-1

**Published:** 2020-07-16

**Authors:** Dong Wook Kwak, Shin Young Kim, Hyun Jin Kim, Ji Hyae Lim, Young-Han Kim, Hyun Mee Ryu

**Affiliations:** 1 Department of Obstetrics and Gynecology, Ajou University School of Medicine, Suwon, Korea; 2 Department of Obstetrics and Gynecology, Cheil General Hospital and Women’s Healthcare Center, Seoul, Korea; 3 Department of Obstetrics and Gynecology, CHA Gangnam Medical Center, Seoul, Korea; 4 Center for Prenatal Biomarker Research, CHA Advanced Research Institute, Seongnam, Korea; 5 Division of Maternal Fetal Medicine, Department of Obstetrics and Gynecology, Institute of Women’s Life Medical Science, Yonsei University College of Medicine, 50-1 Yonsei-ro, Seodaemun-gu, Seoul, 03722 Korea; 6 Department of Obstetrics and Gynecology, CHA Bundang Medical Center, CHA University School of Medicine, 59 Yatap-ro, Bundang-gu, Seongnam-si, Gyeonggi-do 13496 Korea

**Keywords:** Predictive markers, Biomarkers, Genetics research

## Abstract

Elevation of total cell-free DNA (cfDNA) in patients with preeclampsia is well-known; however, whether this change precedes the onset of symptoms remains inconclusive. Here, we conducted a nested case–control study to determine the elevation of cfDNA levels in women who subsequently developed preeclampsia. Methylated *HYP2* (m-*HYP2*) levels were determined in 68 blood samples collected from women with hypertensive disorders of pregnancy, along with 136 control samples, using real-time quantitative PCR. The measured m-*HYP2* levels were converted to multiples of the median (MoM) values for correction of maternal characteristics. The m-*HYP2* levels and MoM values in patients with preeclampsia were significantly higher than in controls during the third trimester (*P* < 0.001, both), whereas those for women who subsequently developed preeclampsia did not differ during the second trimester. However, when patients with preeclampsia were divided based on the onset-time of preeclampsia or 10th percentile birth weight, both values were significantly higher in women who subsequently developed early-onset preeclampsia (*P* < 0.05, both) and preeclampsia with small-for-gestational-age (SGA) neonate (*P* < 0.01, both) than controls. These results suggested that total cfDNA levels could be used to predict early-onset preeclampsia or preeclampsia with SGA neonate.

## Introduction

Hypertensive disorders of pregnancy (HDP) are classified into four categories: chronic hypertension, preeclampsia (PE), PE superimposed on chronic hypertension, and gestational hypertension (GH). PE complicates 2–8% of pregnancies and is one of the leading causes of maternal and fetal morbidity and mortality^1,2^. In recent meta-analyses, aspirin prophylaxis was found to be associated with a significant risk reduction of PE in high-risk patients^3,4^. Therefore, early prediction would be important for proper management.

Many researchers have suggested diverse predictors, such as maternal characteristics (age, body mass index, nulliparity, multiple pregnancy, and previous history of PE), biophysical markers (uterine artery Doppler, blood pressure) or biochemical markers (pregnancy-associated plasma protein-A, vascular endothelial growth factor, placental growth factor, soluble fms-like tyrosine kinase 1, and soluble endoglin) for early prediction of PE^5–11^. Cell-free DNA (cfDNA) circulating in the maternal blood is also a candidate biomarker^12^.

It may be maternal or fetal in origin. Fetal cfDNA is shed from the syncytiotrophoblast as an apoptotic fragment during normal cell turnover, and released into maternal circulation. Although the mechanisms of PE are not completely understood, altered apoptosis is known to be involved in its pathogenesis^13^. Various authors have observed elevations in fetal cfDNA level, during the first and second trimesters, in patients who subsequently developed PE^14–16^, and suggested cfDNA as a predictive marker for early-onset PE or ‘any PE’^17^.

A poorly perfused placenta in patients with PE may release circulating factors into maternal circulation, causing damage to maternal vascular endothelial cells and leading to multi-system dysfunction^17^. The clinical syndrome of PE is a consequence of a wide systemic inflammatory response, and systemic inflammation is associated with the release of cfDNA into circulation^18^. Several studies have shown the amount of total cfDNA to be significantly elevated in patients with PE^19–21^. However, whether total cfDNA is elevated before symptom onset still remains unclear. A few previous studies had shown total cfDNA to be increased in patients with PE during the first or second trimester^22–24^, whereas a recent case–control study of a relatively large sample size demonstrated the total cfDNA levels to not be increased in the first trimester^25^.

The aim of this study was to determine whether the concentrations of total cfDNA in blood are increased in women during the second trimester of pregnancy who subsequently develop HDP during the third trimester. We used the methylated *HYP2* gene as a total cfDNA marker to verify whether total cfDNA levels could be used to predict PE.

## Results

### Clinical characteristics of the study population

Clinical characteristics of the study population are presented in Table 1. There was no significant difference in nulliparity and gestational ages at the time of sampling across the groups (*P* > 0.05 for all). However, body mass index was significantly higher, and gestational age at delivery and birth weight were significantly lower in all patient groups compared to those in the control group (*P* < 0.05 for all). The maternal age of patient groups differed in the second trimester compared to that in the control group, but the difference was not observed in the third trimester.

**Table 1:** Clinical characteristics of the study population.

Characteristics	Controls	PE	GH	*P*^†^	*P*^a^^‡^	*P*^b^^‡^
*Second trimester*	(*n* = 78)	(*n* = 29)	(*n* = 12)			
Maternal age (years)	33.5 ± 3.5	35.5 ± 3.7	36.3 ± 4.3	0.007	0.028	0.029
Nulliparity	9 (29.0%)	8 (27.6%)	4 (33.3%)	0.93	1.00	1.00
GA at sampling (weeks)	21.6 ± 4.2	20.0 ± 3.9	20.0 ± 4.2	0.13	0.13	0.38
BMI at sampling (kg/m^2^)	22.6 ± 2.4	24.2 ± 3.2	26.8 ± 6.0	< .0001	0.03	< .0001
GA at delivery (weeks)	39.5 ± 1.0	37.8 ± 2.6	38.8 ± 1.4	0.003	0.001	0.50
Birthweight (g)	3,245 ± 336	2,785 ± 767	2,961 ± 282	0.008	0.004	0.24
*Third trimester*	(*n* = 58)	(*n* = 20)	(*n* = 7)			
Maternal age (years)	34.2 ± 3.5	34.5 ± 3.4	36.0 ± 2.6	0.41	0.93	0.33
Nulliparity	10 (52.6%)	9 (45.0%)	2 (28.6%)	0.61	0.63	0.63
GA at sampling (weeks)	36.8 ± 0.7	36.4 ± 2.1	37.5 ± 1.5	0.12	0.37	0.30
BMI at sampling (kg/m^2^)	25.6 ± 2.6	28.2 ± 3.5	29.6 ± 4.1	< .0001	0.002	0.002
GA at delivery (weeks)	39.7 ± 1.1	37.3 ± 2.2	39.3 ± 1.1	< .001	< .0001	0.81
Birthweight (g)	3,281 ± 360	2,668 ± 610	3,122 ± 337	0.001	< .001	0.68

Data are given as mean ± SD or number (%).

*P*^a^, PE versus controls; *P*^b^, GH versus controls.

*BMI* body mass index, *GA* gestational age, *GH* gestational hypertension, *PE* preeclampsia.

^†^*P* value by Pearson’s chi-square test, Fisher's exact test or ANOVA as appropriate.

^‡^Adjusted *P* value by Pearson’s chi-square test, Fisher's exact test with step-up Bonferroni method or Dunnett multiple comparisons test.

### Comparison of methylated *HYP2* levels and multiples of the median (MoM) values between women with hypertensive disorders of pregnancy and controls

**Figure 1:**
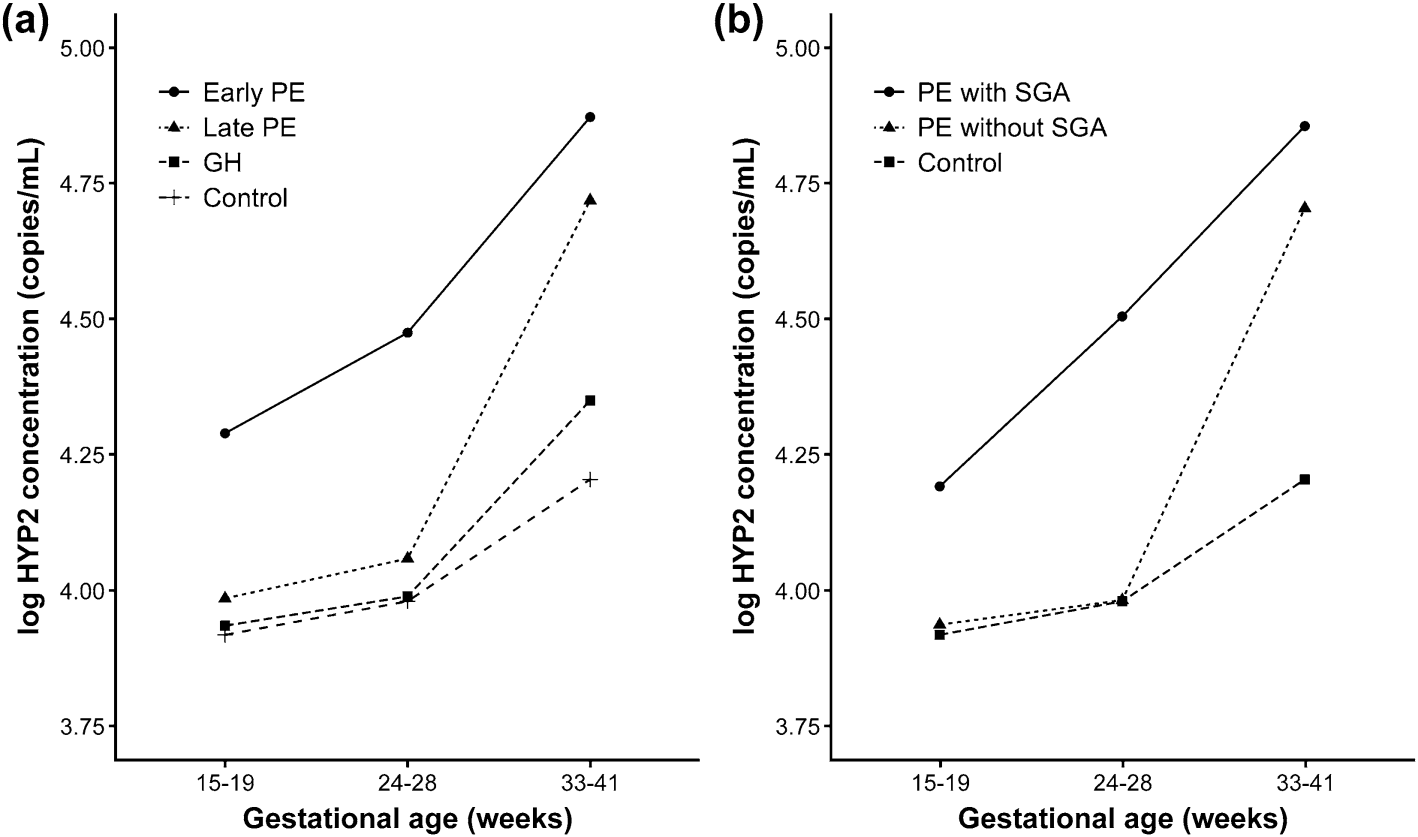
Relationship between total cell-free DNA levels and gestational age at sampling according to (**a**) onset-time of preeclampsia and (**b**) small-for-gestational-age neonate. *GH* gestational hypertension, *PE* preeclampsia, *SGA* small for gestational age.

Comparison of methylated *HYP2* levels in the maternal plasma between specific patient groups and controls is shown in Table 2. The methylated *HYP2* levels and MoM values of the patients with PE were significantly higher than those of normal controls during the third trimester (*P* < 0.0001, both). In contrast, during the second trimester, the methylated *HYP2* concentrations and MoM values of pregnant women who subsequently developed PE, and that of controls, were not significantly different. Nevertheless, when the patients were divided based on the onset time of PE, methylated *HYP2* levels and MoM values were significantly higher in patients with early-onset PE than in control subjects (*P* = 0.042 and 0.044, respectively) (Table 3). Furthermore, when we divided the patients with PE based on the 10th percentile birth weight, both the median methylated *HYP2* concentrations and MoM values were significantly higher in patients who subsequently developed PE, and having a small-for-gestational-age (SGA) neonate, than in patients who subsequently developed PE without SGA neonate (*P* = 0.032 and 0.034, respectively) and in control subjects (*P* = 0.008 and 0.009, respectively) (Table 4). However, these differences were not significant in patients who subsequently developed GH (Table 5). Additionally, we determined the relationship between total cfDNA levels and gestational age at sampling in patients and controls, according to onset-time of PE and accompanying SGA neonate, as shown in Fig. 1. The total cfDNA, in patients with early-onset PE and PE with SGA, was elevated early in the second trimester.

**Table 2:** Comparison of methylated *HYP2* levels and MoM values between specific groups of patients and controls in the second and third trimesters.

	Controls	PE	GH	*P*^†^	*P*^a^^‡^	*P*^b^^‡^	*P*^c^^‡^
*2nd trimester*	(*n* = 78)	(*n* = 29)	(*n* = 12)				
Copies/mL	9,441 (5,878–12,251)	10,964 (6,770–17,509)	8,689 (6,584–11,968)	0.28	0.43	0.70	0.43
MoM	0.998 (0.952–1.026)	1.015 (0.962–1.062)	0.991 (0.946–1.015)	0.29	0.43	0.57	0.43
*3rd trimester*	(*n* = 58)	(*n* = 20)	(*n* = 7)				
Copies/mL	14,969 (11,411–21,873)	59,488 (36,880–87,854)	28,265 (15,330–34,157)	< .0001	< .0001	0.11	0.013
MoM	0.979 (0.948–1.029)	1.090 (1.035–1.149)	0.986 (0.942–1.069)	< .0001	< .0001	0.53	0.032

Data are given as median (interquartile range).

*P*^a^, PE versus controls; *P*^b^, GH versus controls; *P*^c^, PE versus GH.

*GH* gestational hypertension, *MoM* multiple of the median, *PE* preeclampsia.

^†^*P* value by the Kruskal–Wallis test.

^‡^*P* value by the Wilcoxon rank sum test (adjusted by the step-up Bonferroni method).

**Table 3:** Comparison of methylated *HYP2* levels and MoM values between controls and patients with preeclampsia in terms of onset time of preeclampsia.

	Controls	Preeclampsia		*P*^†^	*P*^a^^‡^	*P*^b^^‡^	*P*^c^^‡^
		EO-PE	LO-PE				
*2nd trimester*	(*n* = 78)	(*n* = 6)	(*n* = 23)				
Copies/mL	9,441 (5,878–12,251)	22,394 (14,992–38,659)	10,023 (6,356–16,374)	0.041	0.042	0.63	0.11
MoM	0.998 (0.952–1.026)	1.094 (1.057–1.140)	1.008 (0.951–1.051)	0.044	0.044	0.70	0.10
*3rd trimester*	(*n* = 58)	(*n* = 6)	(*n* = 14)				
Copies/mL	14,969 (11,411–21,873)	72,312 (39,291–131,475)	51,918 (30,462–86,669)	< .0001	0.001	< .0001	0.31
MoM	0.979 (0.948–1.029)	1.103 (1.041–1.118)	1.084 (1.028–1.154)	< .0001	0.008	< .001	0.96

Data are given as median (interquartile range).

*P*^a^, EO-PE versus controls; *P*^b^, LO-PE versus controls; *P*^c^, EO-PE versus LO-PE.

*EO-PE* early-onset preeclampsia, *LO-PE* late-onset preeclampsia, *MoM* multiple of the median.

^†^*P* value by the Kruskal–Wallis test.

^‡^*P* value by the Wilcoxon rank sum test (adjusted by using the step-up Bonferroni method).

**Table 4:** Comparison of methylated *HYP2* levels and MoM values between controls and patients with preeclampsia with or without SGA neonates.

	Controls	Preeclampsia		*P*^†^	*P*^a^^‡^	*P*^b^^‡^	*P*^c^^‡^
		SGA ( +)	SGA ( −)				
*2nd trimester*	(*n* = 78)	(*n* = 11)	(*n* = 18)				
Copies/mL	9,441 (5,878–12,251)	18,503 (10,964–38,959)	9,643 (4,629–12,349)	0.007	0.008	0.69	0.032
MoM	0.998 (0.952–1.026)	1.077 (1.015–1.140)	0.995 (0.909–1.036)	0.008	0.009	0.62	0.034
*3rd trimester*	(*n* = 58)	(*n* = 8)	(*n* = 12)				
Copies/mL	14,969 (11,411–21,873)	68,092 (51,688–103,911)	47,160 (28,833–87,854)	< .0001	< .0001	< .001	0.27
MoM	0.979 (0.948–1.029)	1.086 (1.075–1.116)	1.114 (1.017–1.181)	< .0001	0.002	0.001	0.85

Data are given as median (interquartile range).

*P*^a^, PE with SGA versus controls; *P*^b^, PE without SGA versus controls; *P*^c^, PE with SGA versus PE without SGA.

*MoM* multiple of the median, *SGA* small for gestational age.

^†^*P* value by the Kruskal–Wallis test.

^‡^*P* value by the Wilcoxon rank sum test (adjusted by the step-up Bonferroni method).

**Table 5:** Comparison of methylated *HYP2* levels and MoM values between controls and patients with gestational hypertension with or without SGA neonates.

	Controls	Gestational hypertension (GH)		*P*^†^	*P*^a^^‡^	*P*^b^^‡^	*P*^c^^‡^
		SGA ( +)	SGA ( −)				
*2nd trimester*	(*n* = 78)	(*n* = 4)	(*n* = 8)				
Copies/mL	9,441 (5,878–12,251)	11,800 (8,661–27,875)	8,137 (4,665–9,973)	0.20	0.24	0.24	0.24
MoM	0.998 (0.952–1.026)	1.043 (0.997–1.093)	0.993 (0.911–1.012)	0.11	0.21	0.21	0.21
*3rd trimester*	(*n* = 58)	(*n* = 1)	(*n* = 6)				
Copies/mL	14,969 (11,411–21,873)	31,699 (31,699–31,699)	23,592 (15,330–34,157)	0.21	0.46	0.46	0.81
MoM	0.979 (0.948–1.029)	1.055 (1.055–1.055)	1.014 (0.984–1.047)	0.46	0.70	0.84	0.84

Data are given as median (interquartile range).

*P*^a^, GH with SGA versus controls; *P*^b^, GH without SGA versus controls; *P*^c^, GH with SGA versus GH without SGA.

*MoM* multiple of the median, *SGA* small for gestational age.

^†^*P* value by the Kruskal–Wallis test.

^‡^*P* value by the Wilcoxon rank sum test (adjusted by the step-up Bonferroni method).

## Discussion

This study demonstrated that, during the second trimester, the concentrations of total cfDNA in patients who subsequently developed PE and GH were not significantly different from those in control subjects. However, the elevation became significant when the patient group was limited to early-onset PE or PE with SGA neonate.

Several studies have shown that fetal cfDNA is increased in patients who subsequently developed PE, and the elevation often occurs in the first trimester^15^. However, a few authors have reported the fetal fraction to be low in these patients, as observed during cfDNA screening for aneuploidy^26,27^. Rolnik et al. had indicated that the apparently discrepant results (lower fetal fraction as opposed to increase in absolute quantities of fetal cfDNA) may be due to a less-pronounced increase of fetal cfDNA than of maternal cfDNA, with a consequent reduction in fetal fraction^26^. In a previous study by our group, Kim et al.^24^ had shown that the combination of fetal cfDNA, total cfDNA, and pregnancy associated plasma protein-A can be a useful predictor for PE during the first trimester. In that study, we had demonstrated the total cfDNA levels to be significantly higher in patients who subsequently developed PE at 6–14 and 15–23 gestational weeks, using the *HYP2* gene as a marker of total cfDNA. In contrast, Rolnik et al.^28^ had found a significant increase in the median total cfDNA measured at 11–13 gestational weeks in the early-onset PE group; however, the significance was not observed when the values were corrected for maternal characteristics and gestational age. Furthermore, neither the median total cfDNA nor the MoM values in the late-onset PE group differed from those in controls at 11–13 and 20–24 gestational weeks.

The proportion of early-onset PE is known to be approximately 20%, and around 30% of fetuses born to pregnant women with PE are below the 10th percentile birth weight^29^. Similarly, in our study, the proportion of early-onset PE was 20%, and that of PE with SGA neonate was 37% during the second trimester. Therefore, the low proportion of early-onset PE or PE with SGA neonate may explain the lack of significant differences. In contrast, the opposite result in previous studies may have been due to the higher proportion of such patients in the study population.

Early-onset PE is considered to be somewhat distinct from late-onset PE. The former is typically associated with placental dysfunction, reduction in placental volume, intrauterine growth restriction (IUGR), and adverse neonatal outcomes; In contrast, the latter is more often associated with normal placenta, normal fetal growth, and more favorable outcomes^30^. In this context, several authors have indicated PE as an etiologically heterogeneous disorder that occurs in at least two subsets, one with normal placental function and another involving placental dysfunction^31,32^.

There is a strong correlation between placental dysfunction and fetal growth restriction. IUGR is assigned to infants with a birth weight below 10th percentile for gestational age, having a pathologic restriction on fetal growth due to adverse genetic or environmental influences^33^. Therefore, SGA neonate complicated with PE can be regarded as IUGR. In a prospective study, Milosevic-Stevanovic et al. had shown that placental thickness and weight in patients with PE were significantly different depending on the presence or absence of IUGR. In histopathologic analysis, villous hypermaturity was more frequently observed in the placentas of patients with PE and IUGR^34^. Therefore, the elevation of total cfDNA levels in patients, who subsequently develop PE with IUGR, may be associated with hypermaturation of the placental villi.

The elevation of total cfDNA in patients with PE is thought to be associated with increased neutrophil extracellular trap (NET) production by their neutrophils. NET formation is a defense mechanism in which neutrophils are deployed as an alternative to phagocytosis^35^. Gupta et al. had found that a huge number of NETs was present in the intervillous space of preeclamptic placentae^36^. These NETs appear to be triggered by elevated release of placental micro-debris and may contribute to widespread systemic damage to the maternal endothelium^37,38^. NETs have been identified in both early- and late-onset PE^39^. However, it is currently unclear whether NETs appear prior to the onset of symptoms. Our results implied that, NETs may occur during the preclinical period, although they are limited in preeclamptic patients with placental dysfunction such as early-onset PE or PE with IUGR.

The elevation of total cfDNA may be associated with IUGR due to placental insufficiency regardless of PE. Crowley et al.^40^ had demonstrated that total cfDNA levels are significantly elevated in women with IUGR before 20 weeks of gestation, but not in women with PE. Thereafter, Al Nakib et al.^41^ showed that total cfDNA concentrations are significantly elevated in pregnant women with IUGR due to placental insufficiency, but not due to other causes of IUGR.

The pathophysiology of IUGR is similar to that of PE, and is associated with abnormal placentation, chronic utero-placental ischemia, increased trophoblast apoptosis, and enhanced maternal systemic inflammatory response^42^. In addition, both PE and IUGR promote endothelial cell dysfunction. Formanowicz et al. had reported that the sera collected from women with IUGR and IUGR with PE show a detrimental effect on endothelial cells, reducing their viability and proliferation, and generating oxidative stress owing to dysfunctional mitochondria^43^. Moreover, a few studies had demonstrated cfDNA to serve as an auxiliary biomarker of vascular endothelial dysfunction^44,45^. Therefore, the elevation of total cfDNA before symptom onset, in our patients with early-onset PE and PE with SGA neonate, may be associated with endothelial cell dysfunction. In women with a history of PE, maternal vascular dysfunction may persist for years^46,47^, and the risks of hypertension, cardiovascular disease, stroke, and end-stage renal disease may be increased later in life^48–50^. Yinon et al. had observed reduced flow-mediated dilatation and increased arterial stiffness, in women with previous early-onset PE and in women with previous IUGR without PE, 6–24 months postpartum. In contrast, women with a history of late-onset PE exhibited normal flow-mediated dilatation similar to the control subjects^51^. These findings can be explained by our results. We are not sure whether endothelial damage during pregnancy is the main cause of impaired maternal vascular function in postpartum women. However, a relatively longer period of endothelial cell dysfunction may worsen maternal vascular function in postpartum period, and increased total cfDNA level during the preclinical period, in patients with early-onset PE and PE with IUGR, may indicate the progression of endothelial cell dysfunction.

American College of Obstetrician and Gynecologists guidelines (2018) recommend that low-dose aspirin prophylaxis in women at high risk of PE should be initiated between 12 and 28 weeks of gestation and continued daily until delivery^52^. However, a few studies indicated that aspirin treatment reduces the risk of early-onset PE, but not term PE^53,54^. In this context, prediction of early-onset PE is important, since it may contribute to the identification of women who are most likely to respond to low-dose aspirin. In contrast, low-dose aspirin initiated after 16 weeks of gestation may not be as effective in reducing the risk of PE and fetal growth restriction^3,53^. Therefore, further studies on pregnant women before 16 weeks of gestation would be recommended.

In this study, we included only Korean pregnant women from a single center, and only non-smokers were included in the patient groups. Although this study has limitations its relatively small sample sizes, the homogeneity of our study population may compensate this weakness by minimizing influences from other causes, which can affect total cfDNA levels in maternal blood.

In conclusion, total cfDNA levels were significantly elevated in patients with PE during the third trimester regardless of the onset time of PE or whether the neonate was SGA. However, in the absence of symptoms during the second trimester, elevation of total cfDNA levels was observed only in patients with early-onset PE or PE and SGA neonate. In addition, total cfDNA levels in patients with PE and SGA neonate were significantly higher than in those with PE without SGA neonate. It supports the notion that PE with and without IUGR are two pathogenetically different entities. In future, well-designed studies would be required to confirm the elevation of total cfDNA, in patients with early-onset PE and in patients with IUGR due to placental insufficiency, before 16 weeks of gestation, and to identify the correlation with maternal vascular function in postpartum period. Simultaneously, based on our findings, efforts should continue toward better prediction of PE and IUGR during early pregnancy.

## Methods

### Study participants and samples

We performed a nested case–control study of women with singleton pregnancies who received routine prenatal care at the Department of Obstetrics and Gynecology at Cheil General Hospital between August 2010 and August 2014. This study was approved by the Institutional Review Board and Ethics Committee of Cheil General Hospital (#CGH-IRB-2013-54), and informed consent was obtained from all study participants prior to the study. All experiments were performed in accordance with the relevant guidelines and regulations. Maternal blood samples were prospectively collected when the participants underwent a routine blood test or were admitted for the management of hypertensive disorders of pregnancy at 15–19, 24–28, and 33–41 weeks according to our study protocol. We selected 68 patients, who were diagnosed with PE or GH in our hospital and delivered their baby in the third trimester, and 136 normal controls without medical or obstetric complications. PE was defined as hypertension (systolic blood pressure ≥ 140 mmHg and/or diastolic blood pressure ≥ 90 mmHg, twice, 4 h apart) and proteinuria (≥ 0.3 g/day urine collection and/or ≥ 1 + on dipstick testing) after 20 weeks of gestation. GH is a new-onset hypertension that occurs after 20 weeks of gestation without proteinuria. Early-onset PE was defined as PE diagnosed before 34 weeks of gestation, and late-onset PE was considered if it was diagnosed at or after 34 weeks. SGA was defined as birth weight below the 10th percentile^55^.

### Laboratory analysis

DNA extraction, methylated DNA enrichment, and real-time quantitative PCR were performed as described in our previous study^24^. Maternal blood samples (10 mL) were collected in EDTA tubes and were immediately centrifuged at 1,600×*g* for 10 min at 4 °C. The supernatant plasma was re-centrifuged at 16,000×*g* for 10 min at 4 °C and aliquoted into 1 mL for circulating cfDNA extraction. Circulating cfDNA was extracted using the QIAamp DSP Virus Kit (Qiagen Hilden, Germany). The MethylMiner™ methylated DNA enrichment kit (Invitrogen, Carlsbad, CA., USA) with methyl-CpG binding domain (MBD) biotin protein was used to isolate methylated cfDNA from that extracted from maternal plasma. Finally, the isolated methylated cfDNA was concentrated using a DNA concentrator (Zymo Research Corp., Irvine, CA, USA) and then eluted in a final volume of 30 μL. Enrichment of methylated DNA enrichment was validated using control DNA (methylated and unmethylated DNA) included in the kit according to the manufacturer’s recommendations.

We measured the levels of total cfDNA by real-time quantitative PCR in all samples without failure of MBD capture. Quantification of the methylated *HYP2* gene as an epigenetic marker of total cfDNA was performed in duplex reactions. Real-time quantitative PCR amplification was performed using the ABI 7500 Real Time System (Applied Biosystems, Foster City, CA, USA). Duplex reactions were prepared in a volume of 20 μL, using 5 μL of 4 × NEXpro qRT-PCR Master Mix (Geneslabs, Seongnam, Korea) and 6 μL of the methylated plasma DNA captured by MBD. Primers and probes were both used at a final concentration of 250 nM for *HYP2*. A standard curve using serial dilutions of single-stranded synthetic DNA oligonucleotides specific to the *HYP2* amplicons (Bioneer, Daejeon, Korea) was employed. Each standard was amplified in triplicate and included in every PCR plate. All samples were amplified in triplicate and the final data reflected average of the results.

### Comparison of methylated *HYP2* levels between patients and controls

We converted the measured levels of methylated *HYP2* to MoM values to correct for maternal characteristics, such as gestational age and maternal body mass index at the time of sampling, to increase the statistical power and compared the levels of methylated *HYP2* and MoM values between patient groups (PE and GH) and controls. For further analysis, patients with PE were divided into subgroups according to the onset time of PE or diagnosis of SGA neonate, and the values of each group of patients (i.e. early-onset or late-onset PE, and PE with or without SGA neonate) were compared with those of controls.

### Statistical analyses

Values are presented as frequencies (percentages) or medians (interquartile ranges), as appropriate. The three groups were compared using the Chi-square test or Fisher’s exact test for categorical variables, and the Kruskal–Wallis test were performed to compare continuous variables. If the Kruskal–Wallis test was significant, pair-wise comparisons of the three groups were performed using the Wilcoxon rank sum test with the step-up Bonferroni method. The MoM values of methylated *HYP2* levels were calculated by dividing the expected methylated *HYP2* levels by the actual measured methylated *HYP2* levels. The expected methylated *HYP2* level was calculated by quantile regression, which aimed at estimating the conditional median of dependent variable. In all tests, a threshold of *P* < 0.05 was defined as statistically significant. Statistical analyses were performed using SAS version 9.4 software (SAS, Inc., Cary, NC, USA; https://www.sas.com/) and R 3.4.1 (Vienna, Austria; https://www.R-project.org/).
